# Effects of Isorhamnetin on Diabetes and Its Associated Complications: A Review of In Vitro and In Vivo Studies and a Post Hoc Transcriptome Analysis of Involved Molecular Pathways

**DOI:** 10.3390/ijms23020704

**Published:** 2022-01-09

**Authors:** Feten Zar Kalai, Mondher Boulaaba, Farhana Ferdousi, Hiroko Isoda

**Affiliations:** 1 Alliance for Research on the Mediterranean and North Africa (ARENA), University of Tsukuba, Tsukuba 305-8572, Japan; zarfeten@gmail.com (F.Z.K.); mondher1382@gmail.com (M.B.); ferdousi.farhana.fn@u.tsukuba.ac.jp (F.F.); 2 Laboratory of Aromatic and Medicinal Plants, Center of Biotechnology, Technopark of Borj Cedria, BP 901, Hammam-Lif 2050, Tunisia; 3 Faculty of Life and Environmental Sciences, University of Tsukuba, Tsukuba 305-8575, Japan

**Keywords:** isorhamnetin, quercetin, biological activities, diabetes, molecular pathways, microarray

## Abstract

Diabetes mellitus, especially type 2 (T2DM), is a major public health problem globally. DM is characterized by high levels of glycemia and insulinemia due to impaired insulin secretion and insulin sensitivity of the cells, known as insulin resistance. T2DM causes multiple and severe complications such as nephropathy, neuropathy, and retinopathy causing cell oxidative damages in different internal tissues, particularly the pancreas, heart, adipose tissue, liver, and kidneys. Plant extracts and their bioactive phytochemicals are gaining interest as new therapeutic and preventive alternatives for T2DM and its associated complications. In this regard, isorhamnetin, a plant flavonoid, has long been studied for its potential anti-diabetic effects. This review describes its impact on reducing diabetes-related disorders by decreasing glucose levels, ameliorating the oxidative status, alleviating inflammation, and modulating lipid metabolism and adipocyte differentiation by regulating involved signaling pathways reported in the in vitro and in vivo studies. Additionally, we include a post hoc whole-genome transcriptome analysis of biological activities of isorhamnetin using a stem cell-based tool.

## 1. Introduction

Plants have been used as traditional medicines in almost all continents of the world since ancient times. Scientific research into the reasons for their medicinal uses has led to the exploration of bioactive molecules. Extremophile plants that grow in extreme environmental conditions are considered a good potential source of bioactive molecules of interest. In fact, these environmental constraints are at the origin of dysfunctional oxygen metabolism, which leads to oxidative stress by increasing reactive oxygen species (ROS) [1,2]. Some plants, such as halophytes, have a powerful antioxidant system to eliminate these harmful compounds. Among the bioactive molecules of interest, there are phenolic compounds. Several studies have valued plants rich in polyphenols with or without restrictive conditions in the laboratory [3,4,5,6,7,8,9,10]. Several biological effects are attributed to extracts rich in polyphenols, such as anti-inflammatory and anticancer activities [11,12,13]. Other studies showed the antioxidant, the antimicrobial [8,14,15,16] as well as anti-obesity, anti-diabetic and anti-hepatic steatotic [17,18,19] effects of isorhamnetin. Among these polyphenols, flavonoids are distinguished. This group includes several subgroups, such as flavonols. Isorhamnetin is one of the major compounds of flavonols. Isorhamnetin is a monomethoxyflavone or an O-methylated flavonol from the class of flavonoids. It is quercetin in which a methoxy group replaces the hydroxy group at position 3’. Some isorhamnetin derivatives are present in nature, such as isorhamnetin 3-O-β-d-glucopyranoside, isorhamnetin 3-O-neohesperidoside, and isorhamnetin 3-O-rutinoside from *Calendula officinalis* L. [20]. Isorhamnetin presents significant biological properties such as antioxidant [21], anticancer [22], antimicrobial [23], antiviral [24], anti-inflammatory and anti-diabetic effects [21,25,26,27,28,29,30,31,32,33].

In this review, the first focus is on the origin, chemical structure, isolation and extraction methods, as well as the phytochemical aspect of isorhamnetin. Then, in the second part, we focus on describing the potential anti-diabetic effects of this flavonol through reducing diabetes-related disorders by decreasing glucose levels, ameliorating the oxidative status, alleviating inflammation, and modulating lipid metabolism and adipocyte differentiation. Finally, we performed a secondary analysis of our previously published whole-genome microarray data to explore diabetes-related bioactivities of isorhamnetin in a stem cell-based tool. We also aim to highlight the effect of this molecule on the regulation of the involved signaling pathways by reporting the in vitro and in vivo studies used in this field of research. In this review, we exposed the characteristics of the anti-diabetic activity of isorhamnetin in comparison with quercetin which is considered to be a metabolite of isorhamnetin and an important reference in the natural treatment of diabetes.

## 2. General Overview on Bioactive Molecules in Particular Polyphenols and Flavonoids

### 2.1. Oxidative Stress as an Origin of Bioactive Molecules in Plants

Several plants can be subjected to various environmental conditions (salinity, drought, UV rays, heavy metals, extreme temperatures, nutrient deficiency, air pollution and pathogen attacks). These constraints are at the origin of dysfunctions of oxygen metabolism, which generate oxidative stress by increasing reactive oxygen species (ROS). The oxygen molecule (O_2_) plays an important role in photosynthetic organisms. Originally, in higher plants and algae, gas exchange involving electrons occurs in chloroplasts by capturing carbon dioxide during the day and producing oxygen. This gas exchange involves electrons. Indeed, in the case of severe environmental constraints, a large part of oxygen is not reduced and can thus generate ROS in some organelles from plant cells [1,2] due to an imbalance in the proper functioning of chloroplasts and the transfer of electrons [1]. Moreover, under the environmental constraints as mentioned above, many ROSs are produced, such as hydroxyl radical (^.^OH), the superoxide anion radical (O_2_^.−^), alkoxyl and peroxyl radicals (RO^.^ and RO_2_, respectively), hydrogen peroxide (H_2_O_2_), hypochlorite radical (^−^OCl), singlet oxygen (O_2_), nitric oxide radical (NO) and other lipid peroxides (such as malondialdehyde and 4-hydroxynonenal) [2,34,35]. Sometimes, ROS may have a role in cell signaling in the physiological behavior of plants, for example in the process of seed growth and development, development of tissues, and the transition from cellular proliferation to cell elongation during the earlier stages of differentiation [36]. At a high level, these molecules cause molecular damage such as lipid peroxidation of membranes, alteration of proteins and DNA, and cell death [1,35,37]. Some plants, such as halophytes, have the capacity to adapt well to these conditions through a powerful antioxidant system. Halophytes are known to be a source of secondary metabolites such as polyphenols [3,4,5,6,7,8,9,10]. These authors show that under severe conditions, polyphenols are synthesized to play an important role in protecting against stress-induced oxidative damage. The biosynthesis, the content and the activities of these phenolic compounds are a function of several extrinsic (light, temperature, salinity and dryness) and intrinsic (genotype, organ and stage of development) parameters influencing their content and distribution in plants [8,38,39]. For example, in *Pyracantha coccinea*, some flavonoids as flavanones, flavones, and flavonols are present in the shoots during the vegetative phase and in the roots exclusively during the reproductive one [40]. On the other hand, several authors have shown that these phenolic compounds have other biological properties such as anti-inflammatory and anticancer activities [11,12,13]. Other studies showed the antioxidant, antimicrobial [8,14,15,16] as well as anti-diabetic [18,41,42] effects of phenolic extracts.

### 2.2. Classification of Natural Antioxidants

The antioxidant system can be classified according to the nature of these components into enzymatic or non-enzymatic compounds. The first include superoxide dismutase (SOD), catalase (CAT), ascorbate peroxidase, and glutathione reductase [1,35,42,43,44]. The genes relating to these enzymes revealed their importance during the postharvest physiological deterioration of storage root and in response to osmotic stress and abscisic acid as well as *Xanthomonas axonopodis* infection [44]. The second group contains mainly phenolic compounds, carotenoids, vitamins and osmolytes [1]. Phenolic compounds are characterized by the presence of one or more benzene rings and differ in the complexity of the base molecule, the number and location of the hydroxyl and the degree of polymerization. These compounds are secondary metabolites that fall into three broad groups: phenolic acids (derivatives of benzoic and cinnamic acids), flavonoids (flavonols, flavanols, flavanones, flavones, anthocyanins) and tannins (hydrolyzable tannins and proanthocyanidins). In addition to these molecules, stilbenes, lignans and coumarins are also distinguished [45].

### 2.3. Origins and Biochemical Structure of Flavonoids, in Particular, Isorhamnetin

**Figure 1:**
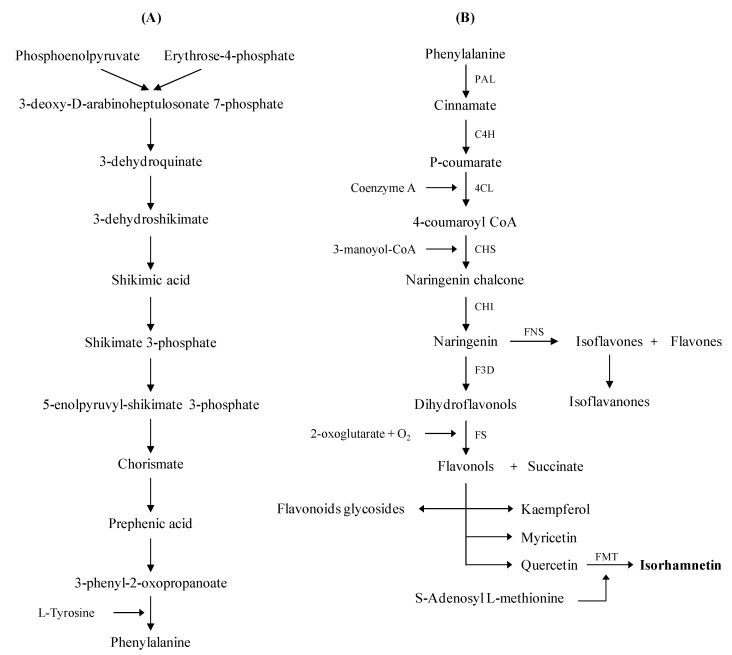
Simplified biosynthetic of isorhamnetin by (**A**) the shikimic and (**B**) the phenylpropanoid pathways. PAL: phenylalanine ammonia lyase, C4H: cinnamate 4-hydroxylase, 4CL: 4-coumaroyl-coenzyme A ligase, CHS: chalcone synthase, CHI: chalcone-flavanone isomerase, FNS: flavone synthase, F3D: flavanone 3-dioxygenase, FS: flavonol synthase, FMT: flavone 3′-O-methyltransferase.

Phenolic compounds are secondary metabolites prevalent in plants. These compounds are with an aromatic ring with one or more hydroxyl groups (OH) and contain molecules ranging from simple phenolic acids to polymerized compounds such as tannins. The synthesis of phenolic compounds is a complex process that goes through several stages. Phenolic compounds are bioactive molecules with two origin pathways: in one side the shikimic acid and on the other side, the phenylpropanoid molecules. The biosynthesis of flavonoids such as isorhamnetin is based on these pathways. In fact, on the one hand, shikimate gives the basic skeleton of polyphenols which have one or more benzene rings (C6) carrying one or more hydroxyl functions. On the other hand, there is the synthesis of the C_6_-C_3_ base formed by the condensation of phenylalanine to cinnamic acid (Figure 1) [46].

More precisely, shikimic acid is at the base of several reactions, representing the skeleton of aromatic amino acids, which are the initiators of phenolic compounds. The first step consists of a combination of two molecules: phosphoenolpyruvate and erythrose 4-phosphate, which after four reactions, lead to forming the first skeleton of phenolics: the shikimate or shikimic acid (C_7_H_9_O_5_). This later presents the first ring, which characterizes phenolics with two hydroxyl groups. The shikimate undergoes six reactions which end in the first amino acid: phenylalanine. Thanks to two key enzymes, phenylalanine ammonia-lyase (PAL) and cinnamate-4-hydroxylase (C4H), phenylalanine successively forms cinnamate and p-coumarate.. In this step, the level of PAL activity could quantitatively regulate the accumulation of phenolic compounds. The *p*-coumarate molecule is at the origin of coumarin derivatives. The pathway for the synthesis of phenylpropanoid molecules is characterized by the presence of a key enzyme called 4-coumarate CoA ligase (4CL), which catalyzes in the presence of the thiol function of the coenzyme A (CoA) the *p*-coumaric acid into 4-coumaroyl CoA (C_30_H_42_N_7_O_18_P_3_S). The aromatic A-ring of flavonoids provides from the condensation of three molecules of malonyl-CoA (-C6).

Subsequently, the 4-coumaroyl CoA produced the naringenin chalcone, explaining the link between the aromatic B-ring and the 3C one of chalcone (C6-C3-). Chalcone is a key element in this topic because it is a precursor of all flavonoids based upon a fifteen-carbon skeleton consisting of two benzene rings. Afterward, chalcone is converted to naringenin (also called flavanone or trihydroxyflavone) by the action of chalcone isomerase (CHI). On the one hand, flavones such as apigenin, acacetin, chrysin or luteolin are synthesized from naringenin in the presence of flavone synthase (FNS). On the other hand, different flavonols and succinic acid compounds are produced from naringenin in the presence of two enzymes: flavanone 3-dioxygenase (F3D) and flavonol synthase (FS). Apart from flavonol derivatives, other compounds, such as kaempferol, myricetin, and quercetin, are also produced. By a methyl group transfer from the S-adenosyl-L-methionine, the isorhamnetin is produced in the presence of the flavone 3′-O-methyltransferase (FMT) [47,48]. Then, isorhamnetin is a monomethoxyflavone or an O-methylated flavonol from the class of flavonoids. It is a quercetin (precursor) in which the hydroxy group at position 3’ is replaced by a methoxy group. Some isorhamnetin derivatives are present in nature, such as isorhamnetin 3-O-β-d-glucopyranoside, isorhamnetin 3-O-neohesperidoside, and isorhamnetin 3-O-rutinoside from *Calendula officinalis* L. [20].

**Figure 2:**
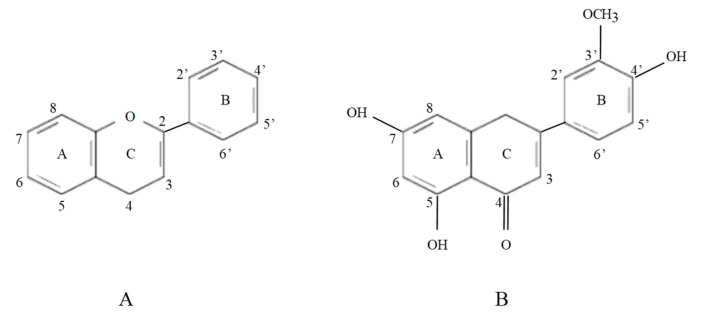
(**A**): Basic skeleton of flavonoids, (**B**): Isorhamnetin.

In fact, flavonoids are considered one of the most important groups of the polyphenol family. They have a structure based on a diphenyl propane type with two benzene rings (ring A and B, see Figure 2) linked by a three-carbon chain that forms a closed pyran ring (C ring). Therefore, their structure is referred to as C6-C3-C6. O-glycosylation positions are C7 in flavones, isoflavones, flavanones, and flavonols, and C3 in flavonols and anthocyanidins. C-glycosylation positions are C6 and C8 in flavones [49]. In addition, the importance of the antioxidant role of phenolic compounds is related to the degree of hydroxylation of the molecule. Flavonoids include the isoflavones, flavones, flavanones and their glycosides and flavonols as isorhamnetin, which is also named 3’-methoxy quercetin and 3-methyl quercetin [50]. In plants, an enzyme, the UDP-dependent glycosyltransferases, is responsible for the glycoside form of isorhamnetin (isorhamnetin 3-O-glucoside). This enzyme utilizes nucleotide diphosphate sugars, usually uridine diphosphate (UDP)-sugars, to transfer the methyl group to the cycle and associate the glycoside function with isorhamnetin [51].

### 2.4. Isolation and Analyses of Isorhamnetin Originated from Medicinal Plants

The distribution of isorhamnetin in medicinal plants is very wide and the methods of extraction and analyses are varied. Isorhamnetin derivatives are particularly wanted. The hydroxyl and methyl groups help in their characterization. Some methods are used to extract isorhamnetin, among them based on fractionation, using chemometric approaches, enzyme, and supercritical fluid extraction (SFE-CO_2_). Firstly, fractionation can be used to simplify the extraction by removing all oil and lipophilic pigments from lipid-containing samples. The defatted sample is also sonicated before maceration in a mixture of methanol and water. Chromatography can then be used for analyses of phenolic compounds, especially flavonoids [49]. For these last compounds, LC-MS is used and both electrospray ionization (ESI) and atmospheric-pressure chemical ionization are frequently applied, and the best sensitivity for flavonoids is generally achieved in the negative-ion mode [49]. For example, the phytochemistry of *Calligonum azel* Maire plant fractions, collected from the Tunisian desert, have been assessed and an ultra-high performance liquid chromatography coupled to quadruple time of flight mass spectrometry UHPLC-ESI-QTOF was used to identify phenolics among them flavones and flavanols, which were the most abundant phenolic compounds identified [52]. More specifically, the presence of isorhamnetin glucoside and isorhamnetin glucosyl-rhamnoside was confirmed as the major compounds in the leaves of the edible halophyte *Mesembryanthemum edule* using LC/ESI-MS/MS technology [3]. This work was followed by others who have used LC-ESI-TOF-MS to characterize many polyphenols; among them, flavonoids were identified from aerial parts of the full flowering stage of halophytes such as *Arthrocnemum indicum* [5], *Tamarix gallica* [16], *Glaucium flavum* [13], and *Salsola kali* [8]. Similar to last works, LC-ESI-TOF-MS and GC-MS profiling of *Artemisia herba-alba* was performed to identify phenolics, such as flavones, flavonols and flavonoid alkaloids [7]. Another work was carried out on *Pancratium maritimum* and the analysis by HPLC-DAD-ESI/MS revealed the presence of flavonoids including flavonol as isorhamnetin with their pentoside and hexoside conjugates such as isorhamnetin di-hexoside [53]. As a non-fatty compound, flavonols could be isolated by hexane. Then, a polar solvent such as ethanol can be used. A study on *Limoniastrum guyonianum* using HPLC showed the presence of many phenolics, among them isorhamnetin-3-O-rutinoside [54].

Far from phenolic extracts, phenolic compounds are also found in oils such as olive oil [55]. These compounds have the ability to protect oils against oxidation and improve the nutritional value of the oil. Isorhamnetin, as a phenolic compound, has also been detected in an oil extract from Tunisian black cumin (*Nigella sativa* L.) seeds obtained with a green solvent such as 2-methyl tetrahydrofuran (MeTHF) as an alternative to petroleum or hexane-based solvent to extract oil-enriched phenolic compounds [10]. The presence of isorhamnetin was approved by HPLC analysis. Analyses using Liquid Chromatography with Diode Array Detector (LC-DAD) revealed a high amount of isorhamnetin ranging between 6.3 and 6.6 (µg/g oil) after oil extraction from black cumin by MeTHF and hexane, respectively. In addition, using H nuclear magnetic resonance (HNMR) and C nuclear magnetic resonance (CNMR), some flavonol glycosides such as kaempferol-3-O-rutinoside (nicotiflorin) and isorhamnetin-3-O-rutinoside (narcissin) were characterized from the aerial parts of *Peucedanum aucheri* Boiss collected in Marivan city, Kurdistan province, Iran [56]. The NMR method detects the property that certain atomic nuclei of the compound interact with a magnetic field. This property, which is to produce magnetic resonance at a certain frequency, provides information on the structure of the molecule. Secondly, mathematical models are chosen to evaluate the efficiency of flavonol extraction, especially to understand the best way to obtain isorhamnetin-3-O-rutinoside [20]. A multivariate factorial analysis was conducted using flowers of *Calendula officinalis*. Linear, quadratic, full cubic and special cubic models were analyzed. At the final full cubic was the most appropriate one, allowing greater efficiency in the extraction of isorhamnetin-3-O-rutinoside by 60%. For this study, the enzymes, Rapidase Maxi Fruit and Viscozyme, were used under some important factors affecting the enzyme activity under supercritical CO_2_ conditions such as pressure, temperature, pH, time and aqueous ethanol solution. Finally, the supercritical fluid extraction method was used to extract phenolic compounds such as flavonols. Many years ago, the supercritical fluid extraction procedure was used on the *Eucalyptus globulus* bark for the first time, using pure and modified CO_2_ with water, ethyl acetate and ethanol [57]. Authors showed that the supercritical CO_2_ combined with ethanol could extract significant amounts of phenolic compounds, including isorhamnetin. HPLC-MS quantification determined some flavonols, such as isorhamnetin-hexoside (0.26 g·g^−1^ of extract) and simple isorhamnetin (14.29 mg·g^−1^ of extract). Ethanol was used as a co-solvent. The quantification was carried out with high-pressure liquid chromatography equipped with a photodiode array detector. Isorhamnetin 3-O-glucosyl-rhamnosyl-rhamnoside, isorhamnetin 3-O-glucosyl-rhamnosyl-pentoside, isorhamnetin 3-O-glucosyl-rhamnoside, and isorhamnetin 3-O-glucosyl-pentoside were the most abundant flavonols extracted from *O. ficus-indica* supercritical extracts. As mentioned before, high-pressure liquid chromatography or HPLC is a commonly used method for analyzing phenolic compounds. In apple juice and pear juice, isorhamnetin 3-O-glucoside was detected. In fact, the separation of the flavonol glycosides in a “Brettacher” apple extract by HPLC and mass spectrometry revealed the presence of two glycoside forms such as isorhamnetin 3-O-glucose and isorhamnetin 3-O-galactoside [58].

## 3. General Overview of Biological Activities of Isorhamnetin

Oxidative stress is an endogenous and exogenous process that plays a role in aging. Oxidative stress occurs due to dysregulation in the antioxidant defences resulting from the imbalanced production of ROS and nitrogen species [59]. Several researchers have shown that long-term exposure to free radicals contributes to the development of chronic diseases, such as cancer [60], diabetes [61], cardiovascular problems [62], and neurodegenerative disease [63]. Therefore, the study of the biological activities of isorhamnetin can be initiated by investigating the involvement of this molecule in the antioxidant phenomenon. Isorhamnetin belongs to the flavonol class known for its antioxidant potential [64,65]. Moreover, several studies have shown that this flavonol has remarkable antioxidant activity, scavenging DPPH radical and ABTS radical, and can inhibit lipid peroxidation [66,67,68]. Wu et al. showed that isorhamnetin and isorhamnetin-3-glucuronide could inhibit the human breast cancer MCF-7 cell proliferation [69]. Wei et al. proved that isorhamnetin inhibits the proliferation of a cervical cancer cell line HeLa [70]. The mechanism of the anti-proliferative effect of isorhamnetin was thoroughly related to cell cycle arrest at the G_2_/M stage by the activation of the ATM-Chk2 pathway. Li and colleagues revealed that isorhamnetin could also inhibit the growth of the gefitinib resistant PC9 cells (PC9-IR) by downregulating *BCL-2* gene expression and PCNA protein expression, inhibiting DNA synthesis and upregulating *P53*, *BAX* and *CASP3* gene expression [22]. Along with this, Aonuma et al. [71] demonstrated that isorhamnetin might efficiently suppress hypertrophy and fibrosis induced by angiotensin II in cardiac tissue by regulating transforming growth factor (TGF-β) pathways. Furthermore, it might have appreciated properties on potential clinical consequences of cardiovascular diseases by the renin-angiotensin system regulation. Isorhamnetin exerted a neuroprotective effect against ischemic injury in mice by reducing infarct volume and *Casp3* activity (a biomarker of apoptosis) and improving neurological function repossession. Likewise, the mice treated with this flavonol showed decreased cerebral edema, enhanced blood–brain barrier function, and upregulated gene expression of tight junction proteins, including *Ocln*, *Zo-1*, and *Cldn-5* [72]. Ishola et al. reported that isorhamnetin ameliorates scopolamine-induced spatial and non-spatial learning and memory impairment in vivo [73]. Likewise, it could reduce malondialdehyde (MDA) and nitrite production by increasing glutathione (GSH) level, SOD, and CAT activities in the prefrontal cortex and hippocampus. The anti-inflammatory effect of isorhamnetin and related mechanisms have been widely studied [21]. Yang et al. [32] demonstrated that isorhamnetin could alleviate LPS-induced acute lung injury by inhibiting *Cox-2* expression in male BALB/c mice [74]. Similarly, the in vivo study of Dou et al. [75] showed that it could alleviate bowel disease by activating PXR and promoting the upregulation of PXR-mediated metabolism of probiotics and the downregulation of nuclear factor-kappa B (NF-κB) signal transduction [75].

**Figure 3:**
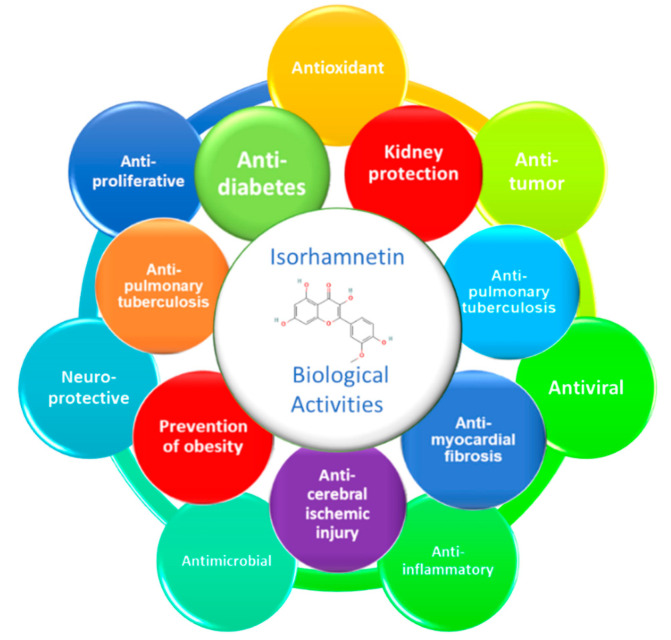
Overview of biological activities of isorhamnetin. NB. Circles do not represent any hierarchical relationship.

Furthermore, isorhamnetin has been reported to prevent pulmonary tuberculosis [76]. Additionally, it may ameliorate acute kidney injury by inhibiting the NF-κB signaling pathway activation [31]. Additionally, isorhamnetin exhibits anti-thrombus [77], antihypertensive [78], anti-inflammatory [26], anti-osteoporosis [79], antiplatelet activity [80], hepatoprotective [81], and anti-hypoxia [82] effects. It has also been shown that isorhamnetin could boost innate immunity [83]. However, we have not found a wide range of publications that confirm antimicrobial and antiviral activities of isorhamnetin. Still, some studies reported bactericidal effects of plant extracts containing isorhamnetin or its derivatives [84,85,86]. Antiviral activities of isorhamnetin were observed against influenza [87], SARS-CoV-2 spike [24], and herpes simplex [88] (see the summary in Figure 3). In summary, this interesting molecule is an immense source of biological activities. More than what we have mentioned, it is endowed with a critical anti-diabetic activity that we study in the next section of this review.

## 4. Anti-Diabetic Effect of Isorhamnetin

Isorhamnetin has been reported to alleviate shared metabolic complications in type 1 diabetes mellitus (T1DM) and type 2 diabetes mellitus (T2DM), but until the present, it is more aligned with T2DM. For example, Matboli et al. showed that isorhamnetin used at three different doses (10, 20, 40 mg/kg for 3 weeks) possessed anti-diabetic action by regulating the insulin pathway at the microscopic, molecular and protein levels in the streptozotocin/high fat diet-induced T2DM rat models [89]. Authors in this study concluded that isorhamnetin could be used as an alternative and/or potential complementary treatment in T2DM through modulation of insulin resistance signaling pathway-related gene expression. However, regarding T1DM, the effect of isorhamnetin is not yet well studied with in vitro and in vivo models.

### 4.1. General Overview on Diabetes and Its Link with Metabolic Syndrome

Metabolic syndrome is a constellation of cardiometabolic risk factors first described 90 years ago [90]. In 1988, the notion of syndrome X appeared [91]. It includes abdominal obesity, insulin resistance, glucose intolerance (or hyperglycemia), hypertension, and dyslipidemia. The combination of these risk factors is linked to an increased risk of developing T2DM and cardiovascular diseases. Diabetes mellitus (DM) is a prevalent metabolic disease characterized by abnormally elevated blood glucose levels. It is classified into type 1 (T1DM) and type 2 (T2DM). The first category of diabetes (T1DM) is typically associated with failure in insulin production resulting from the destruction of pancreatic β-cells by T-cell-mediated autoimmunity. However, T2DM is more specific to insulin resistance and function [92]. The causes of metabolic syndrome are generally poorly understood but involve genetic factors linked to the environment. Among the genetic factors, one can quote those determining the corpulence, the distribution of the fatty mass, the hyperinsulinemia, and various metabolisms (lipoproteins) [93]. The constitutive elements of these factors tend to derive from multivalent logic. The environmental factors, the sedentary lifestyle, smoking, excess calories provided in the form of lipids, and added sugars, in particular, are better known. Many other factors have been recently discovered, such as the presence of inflammatory cells in adipose tissue and alterations in the secretion of adipokines [93]. It is, therefore, necessary to find an adequate and safe treatment given the severe consequences of this disease on health, including diabetic nephropathy, retinopathy, neuropathy, cardiovascular diseases, and diabetic foot ulcer [92].

### 4.2. Effect of Isorhamnetin on Associated Metabolic Pathways

Several in vitro and in vivo studies showed that isorhamnetin might modulate carbohydrate metabolism and exhibit anti-diabetic activities by involving different mechanisms. Researchers have suggested that isorhamnetin promotes carbohydrate metabolism from the stages of digestion and intestinal absorption [94], improves glucose uptake by the liver and muscles [19,95], protects the pancreatic β-cells and alleviates insulin secretion impairment [96]. In addition, isorhamnetin regulates adipose tissue differentiation, growth and maturation [29]. For anti-diabetic potential, researchers have investigated isorhamnetin isolated from different medicinal plants. In Table 1, we have listed the plant sources and extraction procedures of isorhamnetin that regulated diabetes-associated different intracellular signaling pathways. In the rest of this review, we will try to explain the impact of this molecule and its metabolic precursor (quercetin) [97] on the diabetes-associated mechanisms.

#### 4.2.1. Effect of Isorhamnetin on Glucose Transporters

Glucose transporters (GLUT) are a large group of membrane proteins that transport glucose from epithelial cells to blood and from blood to cells passing the intestinal barrier in the gradient direction by passive transport. Some of them are insulin-dependent, notably GLUT4. It is a major insulin-regulated glucose transporter and coordinator of insulin action in adipose. In fact, it is well established that insulin, which enhances glucose uptake, also stimulates the recruitment of GLUT4 to the cell membrane of insulin-sensitive tissue. In a study underlying the anti-diabetic properties of quercetin and isorhamnetin, the authors proved that physiological concentrations of isorhamnetin promoted GLUT4 translocation to the plasma membrane in L6 myotubes through different mechanisms without altering GLUT4 expression [95]. In fact, isorhamnetin could activate the JAK-STAT signaling pathway at 1 nM and 10 nM, allowing glucose transporter translocation induction. JAK-STAT is a signal transduction system composed of a transmembrane receptor, coupled to a Janus Kinase (JAK) enzyme and a STAT-type protein. When a ligand binds to the receptor, it changes its conformation, activating the enzyme JAK, which phosphorylates the STAT protein, inducing a transduction cascade and activating transcription of specific genes [100]. In an in silico study, Selvaraj [101] showed that certain flavonoids, including isorhamnetin, can strongly interact with the active site of GLUT4 protein through H-bond interaction. This finding lets us look for the influence of this molecule on glucose transporters, particularly GLUT 4. By comparison, quercetin stimulates the insulin and AMPK-dependent pathway in the same L6 cells. In fact, Eid et al. have shown that an 18 h treatment of cultured rat L6 skeletal muscle cells with a dose of 50 µM quercetin could stimulate AMPK and increase GULT4 translocation [102]. Likewise, quercetin demonstrated divergent effects on insulin-mediated GLUT4 translocation in adipocytes under basal and insulin-resistant conditions related to its regulation of AMPK activity [103]. Moreover, Eid et al. confirmed that quercetin aglycone, extracted from *Vaccinium vitis* berries, could enhance muscle cell glucose uptake [98]. Guided fractionation of berry extract based on glucose uptake in muscle cells demonstrated that quercetin-3-O-glycosides were the main active compounds. At 50 μM, these compounds enhanced basal glucose uptake by up to 59% following an 18 h treatment, an effect significantly greater than that of 100 nM insulin.

#### 4.2.2. Effect of Isorhamnetin on Peroxisome Proliferator-Activated Receptors (PPARs)

PPARs belong to the transcription nuclear receptor factors class II family with an important action in the glucose and lipid metabolism regulation [104]. They are expressed in various cell types: pancreatic cells, hepatic cells, and adipose cells, thus controlling a wide range of biological processes by the modulation of related genes’ expressions [105]. In mammalian cells, three isoforms are present: PPARα, PPARβ/δ and PPARγ, which regulate genes implicated in glucose and lipid transport, synthesis and fatty acid oxidation [106,107]. Many clinical studies are targeting PPARs for metabolic disorders and diabetes treatments. Thiazolidinediones (TZDs: pioglitazone and rosiglitazone) are a class of oral anti-diabetic drugs that reduce high blood sugar levels and improve the lipid profile of T2DM patients. A huge number of hypotheses suggest that the anti-diabetic effect of TZDs is exerted via PPARγ. Fibrates that target PPARα have also been used to treat hyperlipidemia and diabetes. Isorhamnetin has been reported to exert similar effects as the mentioned drugs, mainly as PPARs antagonists or agonists [28], which ameliorates metabolic complications induced by a high-fat diet or leptin deficiency [33]. Pre-adipocyte cell line model 3T3-L1 cells are widely used in obesity and diabetes research [108,109]. Obesity-induced diabetes is characterized by hypertrophy and hyperplasia of adipocytes. Pretreatment of 3T3-L1 cells with different doses of isorhamnetin could inhibit adipocyte differentiation by decreasing triglyceride (TG) accumulation and glycerol-3-phosphate dehydrogenase (GPDH) activity [29]. At the molecular level, isorhamnetin could also regulate the mRNA expression of the major adipocyte markers, mainly the transcription factors PPARγ and CCAAT/enhancer-binding protein-α (C/EBPα), the master co-working regulators of adipogenesis and cell differentiation [29,30]. These findings were also concordant with studies revealing that *Nitraria retusa* isorhamnetin-rich extract could reduce the fat accumulation in 3T3-L1 adipocytes in a dose-dependent manner [18]. The single-molecule isorhamnetin could also significantly inhibit the differentiation rate of 3T3-L1 cells, leading to reducing lipid droplet content by reducing cell size and number. In addition, oral administration of the *Nitraria retusa* extract could regulate *PPAR*γ and lipogenic enzymes target genes *LPL* and *FAS* [17]. Importantly, a recent study revealed that isorhamnetin could significantly reduce fat amount in animal body via PPARα-dependent pathway [27].

#### 4.2.3. Effect of Isorhamnetin on Hepatic Enzymes

The liver is an organ that plays a key role in carbohydrate homeostasis [110]. Beyond the micro and macrovascular complications of diabetes, the T2DM patient presents more certain hepatic complications with, in the first place, hepatic steatosis. In T2DM, the risk of progression to fibrosis and hepatic inflammation is greater [111], exposing a certain number of diabetic patients to significant clinical consequences, particularly with hepatocarcinoma [112]. In parallel, hepatic complications in diabetic patients have been associated with a higher risk of cardiovascular events, which may have practical consequences in terms of optimization of cardiovascular prevention [113]. We focus on the most important liver complications related to diabetes: fatty liver diseases, hepatic fibrosis, and hepatocellular carcinoma (HCC).

##### Non-Alcoholic Steatohepatitis

Fatty liver disease or non-alcoholic steatohepatitis (NASH) represents a potential hepatic complication. It is characterized as an abnormal accumulation of TGs inside liver cells. In type 2 diabetes, insulin resistance leads to the accumulation of toxic fatty acids. When this accumulation occurs in liver cells it causes non-alcoholic fatty liver disease (NAFLD) and in severe forms steatosis or NASH [111,114]. Ganbold et al. have shown that isorhamnetin ameliorated hepatic steatosis and fibrosis in a NASH mouse model exhibits similar features of human NASH [19]. In fact, compared to the control mouse group, liver samples from the NASH-induced group showed acute fat accumulation overtaking 37% of the oil red O positive area. However, isorhamnetin treatment in the NASH group could reduce oil red O stained area to 22%. In addition, isorhamnetin treatment alleviated NASH-induced gene expression alteration. The authors have shown that isorhamnetin efficiently improved the altered lipid metabolic process in NASH and inhibited de novo lipogenesis.

##### Hepatic Fibrosis

Hepatic fibrosis is excessive scarring resulting from the build-up of connective tissue in the liver. It is caused by excessive production and/or insufficient degradation of extracellular matrix. There are many causes of liver fibrosis. Among them is NASH origin. When there is resistance to insulin, fatty acids build up in the liver, causing toxicity and inflammation. This is accompanied by the production of cytokines and reactive oxygen species. In fibrosis, cytokines are responsible for the activation of hepatic stellate cells (HSCs) and will activate an excessive production of collagen fibers [115,116]. The trigger is chronic aggression, especially in the event of an inflammatory component [115]. TGF-β is a crucial mediator of HSC activation and extracellular matrix accumulation, leading to fibrosis [117]. Therefore, blocking this pathway could be a potential strategy for liver fibrosis [118]. In this context, Yang et al. demonstrated that isorhamnetin inhibited HSC activation and prevented TGF-β-induced expression of fibrogenic genes, including *α-SMA*, *PAI-1*, and *COL1A1* [74]. In fact, the authors revealed that the inhibitory effect of this flavonol was a result of the canonical TGF-β/Smad signaling pathway inhibition. Additionally, isorhamnetin activated Nrf2-ARE signaling and suppressed TGF-β-mediated ROS production, which also contributed to the inhibition of fibrogenic gene expression. Furthermore, isorhamnetin significantly suppressed carbon tetrachloride (CCl4)-induced fibrosis in mice. Liu et al. also showed that isorhamnetin could alleviate CCl4 and bile duct ligation (BDL)-induced liver fibrosis by inhibiting TGF-β1 production and HSC activation [119]. Likewise, Lee et al. demonstrated that isorhamnetin, extracted from *Oenanthe javanica*, could reduce fibrosis in rat HSCs by blocking the extracellular signal regulated kinase (ERK) signaling pathway and inhibiting the proliferation and collagen synthesis of HSC-T6 cells [99]. In fact, pretreatment of HSC-T6 cells with this flavonol repressed serum-induced ERK phosphorylation, the same way as a MEK inhibitor (PD98059).

##### Hepatocellular Carcinoma

Hepatocellular carcinoma is often discovered late at the metastasis stage (in 64% of cases); the most frequent secondary locations are the lung, lymph nodes, kidneys, and adrenal glands [120]. As mentioned above, with the high accumulation of fatty acid in liver cells, physiological disturbances appear over time such as NASH, then inflammation (with production of cytokines and ROS), then in more advanced cases cirrhosis and in severe forms carcinoma [121,122]. The role of isorhamnetin is deduced by its anti-tumor potential activities. Isorhamnetin, extracted from *Hippophae rhamnoides* L., showed potent anti-tumor activity in hepatocellular carcinoma BEL-7402 cells, exerting strong cytotoxicity with IC_50_ at the concentration of 74.4 ± 1.13 µg/mL and 72 h incubation [123]. Cytotoxicity of this flavonol on cancer cells depends on isorhamnetin cellular accumulation. Instead, the pibenzimol hydrochloride staining of isorhamnetin-treated cells displayed condensed and fragmented nuclei and apoptotic bodies. Furthermore, flow cytometry analysis used to evaluate cell cycle progression and differentiate between apoptosis or necrosis, revealed that 13.77% of isorhamnetin-treated BEL-7402 cells (with 50 µg/mL for 48 h) appeared in the hypodiploid peak. Similar work by Wei et al. reported that isorhamnetin inhibited the cell cycle progression of the cervical cancer cell line HeLa, by arresting the G_2_/M phase via activating the ataxia-telangiectasia mutated Chk2 pathway [75]. In another work and by making a comparison, isorhamnetin-3-O-glucoside, quercetin 3-O-rhamnoside-7-O-glucoside and kaempferol-3-O-glucoside-7-O-rhamnoside, extracted from *Cleome droserifolia*, reduced HepG2, a liver hepatocellular carcinoma cell, cell viability and anchorage-independent cell growth in a dose- and time-dependent manner but with a highest effect with the quercetin derivative. Furthermore, they exhibited a reduced migration capacity of HepG2 cells [124].

#### 4.2.4. Effect of Isorhamnetin on Pancreatic β-Cell Dysfunction

Insulin is a hormone naturally secreted by the body that helps our cells, particularly muscle cells, liver cells, adipose cells, and brain cells, absorb glucose from food to be converted into energy for all biological processes and functions [125,126]. In normal conditions, insulin is secreted by pancreatic β-cells, then released into the bloodstream. β-cell dysfunction is characterized by impaired insulin secretion, the critical feature of T2DM, both in its onset and progression [127]. Metabolic stress-induced β-cell dysfunction in T2DM can also be associated with high levels of saturated fats in obesity, leading to increased inflammation and oxidative stress within β-cells. Therefore, impairment of insulin secretion is more severe than insulin resistance [128]. Taken together, new anti-diabetic therapeutic strategies are needed to prevent or recover pancreatic β-cell dysfunction. In this context, Wang et al. suggested that isorhamnetin, derived from *Vernonia anthelmintica* herb, could suppress the proliferation of pancreatic cancer cells (colorectal adenocarcinoma cell line) via arresting the cell cycle in the S phase, which may be an alternative way to prevent pancreatic carcinoma and its metabolic complications [129]. Consistently, another report has shown that isorhamnetin and a glycosylated form of isorhamnetin (isolated from *Salicornia herbacea* plant) could promote glucose-stimulated insulin secretion in insulin-secreting rat insulinoma (INS-1) pancreatic β-cells without affecting cell viability [96]. Moreover, it could stimulate insulin secretion via phosphorylation of total ERK, insulin receptor substrate-2 (IRS-2), phosphatidylinositol 3-kinase (PI3K), Akt, and activated pancreatic and duodenal homeobox-1 (PDX-1) [96]. The enhancement of these markers may alleviate the pancreatic cell dysfunction causing diabetes.

Furthermore, Grdović et al. reported that isorhamnetin originated from the *Castanea sativa* plant species has a protective effect on streptozotocin-induced oxidative damage and β-cell apoptotic death [130]. This beneficial effect was correlated strongly to the antioxidant potential of this molecule by alleviating the cellular biomarkers of oxidative stress, i.e., MDA and intracellular GSH levels, and enhancing the activities of antioxidant enzymes SOD and CAT. The effect was also confirmed at the molecular level by the decreased NF-kB transcription factor stimulated by cell oxidative stress, and thus suggestive of a protective effect within β-cells. Consistently, quercetin, which is metabolized in isorhamnetin, could reduce glucose levels by ameliorating insulin secretion in the streptozotocin-induced type 1 diabetes model in rats [131]. In the same animal model, quercetin showed a protective effect on β-cell integrity by reducing oxidative stress markers [132]. It could also regenerate the islets in pancreatic cells [133].

#### 4.2.5. Effect of Isorhamnetin on NF-κB

NF-κB is a protein of the superfamily of transcription factors involved in the immune and cellular stress responses and is considered as the major mediator of the secretion of inflammatory markers such as cytokines, chemokines, and different key enzymes [130,134,135]. The role of NF-κβ in the pathophysiology of diabetes and its linked vascular complications has been investigated [136]. Hyperglycaemia activates this nuclear factor that in turn induces the secretion and over-expression of pro-inflammatory cytokines, mainly TNF-α, interleukins, TGF-β, and Bcl2, causing vascular complications such as neuropathy, nephropathy, retinopathy, and cardiomyopathy [137]. Many experimental investigations on the preventive effect of flavonols on diabetes-induced vascular dysfunction have demonstrated that isorhamnetin could inhibit the activation of NF-κβ through inducing the activity of pancreatic antioxidant enzymes [138]. Another study reported the inhibitory effect of isorhamnetin on iNOS expression and NO production in activated macrophages, leading to the blocking of NF-κβ [139]. Moreover, Qiu et al. have studied the renoprotective effect of the flavonol isorhamnetin in the T2DM rat model through the modulation of the NF-κβ signaling pathway [31]. Authors have shown that this molecule inhibited the NF-κB signaling activity by increasing the production of NF-κB p65, phospho-NF-κB p65, and phospho-IκBα and inflammatory mediators TNF-α, IL-1β, IL-6, ICAM-1, and TGF-β1, as well as decreasing the NF-κB p65 DNA-binding activity. In addition, it could alleviate oxidative cell stress by regulating MDA and SOD levels, leading to recovery of renal damages in diabetic rats.

## 5. A Post Hoc Transcriptome Analysis Predicts the Potential Effect of the Isorhamnetin on Diabetes in a Stem Cell-Based Tool

We have established a stem cell-based tool using a perinatal stem cell, human amniotic epithelial cells (hAECs), to evaluate the bioactivities of natural compounds employing whole-genome microarray analysis [71,140,141,142,143,144]. In recent years, an increasingly large number of bioactive compounds of medicinal plants have been screened for their potential therapeutic and preventive effects. In this context, stem cell-based approaches using human pluripotent stem cells (hPSCs) receive great attention as physiologically more relevant in vitro human models for drug screening and validation of thousands of compounds in both academic research and the pharmaceutical industry [145,146,147]. However, hPSCs, including embryonic stem cells (hESCs) and induced pluripotent stem cells (hiPSCs), have limited cell resources, require invasive extraction procedures, expensive cell reprogramming, and critical maintenance procedures, as well as pose ethical constraints, and therefore are less favorable as a practical source for drug screening. On the other hand, hAECs are derived from discarded term placenta, a medical waste product. They do not require invasive harvesting procedures and have minimum ethical concerns. Furthermore, hAECs are derived from the pluripotent epiblasts and thus maintain ESC-like multilineage differentiation potential and can be differentiated into cells from all three germ layers [148,149,150,151].

It is worth noting that upon appropriate differentiation protocol, hAECs can be differentiated into hepatocyte-like cells [152,153,154,155], cholangiocyte [156], and, most importantly, pancreatic β-like insulin-producing cells [157,158,159,160]. Transplantation of hAEC-induced pancreatic cells into streptozotocin-induced diabetic mice could normalize the blood glucose level [161]. hAECs [162], as well as exosomes derived from hAECs [163], could accelerate diabetic wound healing via promoting angiogenesis and fibroblast function and reducing inflammation. Incorporating hAECs into islet organoids [164] and shielding native islets with a layer of hAECs [165] could enhance islet engraftment and revascularization in diabetic mice models. Additionally, hAEC-derived hepatocyte-like cells, as well as hAEC itself, have been reported to have therapeutic efficacy in liver diseases, including hepatic fibrosis [166,167], cirrhosis [168], and hepatic failure [169].

**Figure 4:**
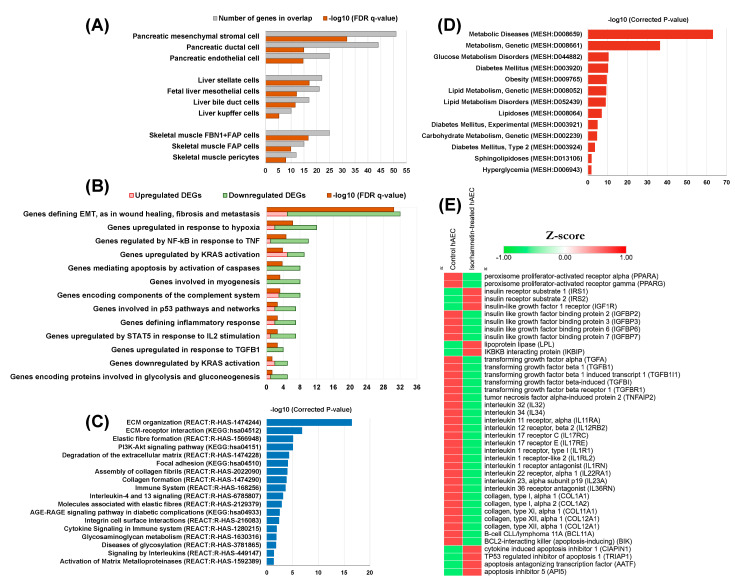
Whole-genome microarray analysis predicts the potential effect of the isorhamnetin on diabetes in a stem cell-based tool of hAEC. (**A**) Significantly enriched cell type signature gene sets (MSigDB of GSEA; https://www.gsea-msigdb.org/gsea/index.jsp, accessed on 26 November 2021); (**B**) significantly enriched hallmark gene sets (GSEA); (**C**) significantly enriched pathways (CTD; http://ctdbase.org/, accessed on 29 November 2021); (**D**) significantly enriched metabolic diseases (CTD); (**E**) heatmap for DM-associated gene expression. All data are available at Gene Expression Omnibus (GEO) under accession number: GSE153149 (https://www.ncbi.nlm.nih.gov/geo/query/acc.cgi?acc=GSE153149, accessed on 24 November 2021).

Considering the complex pathophysiology of DM, hAEC may not be an ideal in vitro model to study the anti-diabetic effects of compounds. However, because of its stem cell-like properties, it can be used for the initial screening of target compounds. We have previously explored antifibrotic [71] and hepatic differentiation-inducing [170] potential of isorhamnetin in hAECs. In the present study, we have performed a targeted secondary analysis of our previously published data [71] to explore the potential functionalities of isorhamnetin in diabetes (Figure 4). Data analysis was conducted for three biological replicates of day 10 control (*n* = 3) and isorhamnetin-treated (*n* = 3) hAECs. The cells were grown on 3D cell culture. Control cells were maintained in placental basal epithelial cell medium (Promo Cell, Cat. #C-26140) in absence of any differentiation medium or growth factors, whereas treatment cells were supplemented with 20 mM of isorhamnetin (Sigma-Aldrich, Japan) for 10 days. Differentially expressed genes (DEGs) are referred to as genes with a linear fold change > 2 and *p*-value < 0.05 (one-way between-subjects ANOVA). A total of 303 DEGs were identified; among them, 60 were upregulated and 243 were downregulated. Details of methodology have been explained elsewhere [71,170]. All microarray data are available at Gene Expression Omnibus (GEO) under accession number: GSE153149 (https://www.ncbi.nlm.nih.gov/geo/query/acc.cgi?acc=GSE153149, accessed on 24 November 2021).

### 5.1. Cell Type Signature Gene Sets

In our previous studies on hAECs, we found that different types of compounds could direct the differentiation of hAECs towards different cell lineages, such as a caffeic acid ester, rosmarinic acid [142] and a caffeoylquinic acid derivative 3,4,5-Tri-O-Caffeoylquinic Acid (TCQA) [140] which could enhance neural cell differentiation, whereas an anthocyanin, cyanidin-3-O-glucoside (Cy3G), induced adipocyte differentiation [143] in hAECs. We observed that the bioactivities or functionalities of natural compounds could generally be predicted from the enriched cell types by the DEGs.

We examined the significantly enriched cell type signature data sets using the Molecular Signatures Database (MSigDB) ver. 7.4 of GSEA online software (https://software.broadinstitute.org/gsea/index.jsp; accessed on 26 November 2021) [171]. These gene sets contain cluster marker signature genes for cell types identified in human tissue single-cell sequencing studies and facilitate the cell type assignments in data sets, such as experiments of developing organoid models.

We found that the most significantly enriched cell type gene set was pancreatic mesenchymal stromal cells [172] (Figure 4A). Additionally, pancreatic ductal and endothelial cell types were significantly enriched [172]. Pancreatic signature genes in isorhamnetin-treated hAECs are involved in epithelial-mesenchymal transition, TGF-β signaling, TNF-α signaling via NF-kB, KRAS signaling, and fatty acid metabolism. Several hepatic signature gene sets were also significantly enriched, such as HSCs, kupffer cells, bile duct cells [173], and fetal liver mesothelial cells [174]. There were also several significantly enriched skeletal muscle signature gene sets, including fibrillin 1^+^fibro-adipogenic progenitor (FBN1^+^FAP) cells, fibro-adipogenic progenitor (FAP) cells, and skeletal muscle pericytes [175]. Biological functions of hepatic signature genes in isorhamnetin-treated hAECs include several inflammatory response pathways, whereas skeletal muscle signature genes regulate wound healing, collagen fibril organization, and MAPK cascade. Significance was measured as the false discovery rate, an analog of hypergeometric *p*-value after Benjamini and Hochberg correction for multiple hypothesis testing (FDR q-value < 0.05).

### 5.2. Significantly Enriched Hallmark Gene Sets

Next, we examined the significantly enriched hallmark gene sets on MSigDB (retrieved on 26 November 2021). Hallmark gene sets represent specific, well-defined biological states or processes generated based on identifying gene set overlaps and retaining genes that display coherent expression. The hallmarks have a collection of 50 gene sets condensed from over 4000 overlapping gene sets and thus have reduced noise and redundancy [176].

Significantly enriched hallmark gene sets include genes defining epithelial-mesenchymal transition, genes upregulated in response to hypoxia, genes regulated by NF-kB in response to TNF, genes up and downregulated by KRAS activation, genes mediating apoptosis by activation of caspases, genes involved in myogenesis, genes upregulated in response to TGF-β1, genes upregulated by STAT5 in response to IL-2 stimulation, genes defining inflammatory response, genes involved in p53 pathways and networks, and genes encoding proteins involved in glycolysis and gluconeogenesis (Figure 4B). Significance was considered at FDR q-value < 0.05.

An interesting finding is enrichment of KRAS activation by the DEGs of the isorhamnetin-treated hAECs. Several KRAS-induced gene expressions were found to be significantly downregulated by isorhamnetin, such as *MMP9*, *TSPAN1*, and *ITGBL1*. Hyperglycemia triggers genomic instability leading to KRAS mutations in pancreatic cells [177] and has also been associated with increased risk and invasiveness of pancreatic [178] and colon cancers [179]. Wang et al. reported that isorhamnetin suppresses proliferation of pancreatic adenocarcinoma cell line PANC-1 through downregulating Ras/MAPK signaling pathway activity [134]. Therefore, as mentioned in Section 4.2.4, the effect of isorhamnetin on the KRAS-induced risk of cancers in diabetes, especially pancreatic cancers, is worth further exploration.

### 5.3. Significantly Enriched Pathways

Further pathway analysis of the DEGs was conducted using the Comparative Toxicogenomics Database (CTD) (http://ctdbase.org/; accessed on 29 November 2021) [180]. CTD represents Kyoto Encyclopedia of Genes and Genomes (KEGG) and REACTOME pathways. We found that several inflammatory pathways, collagen formation and assembly, PI3K-Akt signaling pathway, and AGE-RAGE signaling pathway in diabetic complications were significantly enriched (Figure 4C).

Advanced glycation end products (AGEs) are produced through the non-enzymatic glycation and oxidation of proteins, lipids, and nucleic acids. The receptors for advanced glycation end products (RAGE) belong to the immunoglobulin superfamily. AGE/RAGE signaling is a complex and intricate cascade that activates multiple intracellular signal pathways involving protein kinase C, NADPH oxidase, and MAPKs, resulting in NF-κB -induced expression of IL-1, IL-6, TNF-α, VCAM-1, and VEGF. Particularly, AGE/RAGE signaling has been implicated in diabetes-mediated vascular calcification through activation of TGF-β mediated fibrosis, NFκB, and ERK1/2 pathways [181,182,183,184]. We found that isorhamnetin significantly decreased AGE/RAGE signaling-related gene expression, such as *COL1A1*, *COL1A2*, *COL4A6*, *FN1*, *MMP2*, and *SERPINE1*. As discussed in the earlier section, isorhamnetin’s antifibrotic effects have been well documented [71,74,99,119], and therefore, it can be asserted that isorhamnetin may also have beneficial effects in diabetes-induced vascular pathology.

### 5.4. Significantly Enriched Metabolic Diseases and Related Gene Expressions

Curated gene–disease association data were retrieved from the CTD (retrieved on 29 November 2021). We curated only significantly enriched metabolic diseases. The significance of enrichment was calculated by the hypergeometric distribution adjusted by the Bonferroni method. Significantly enriched metabolic diseases included DM, glucose and lipid metabolism disorders, hyperglycemia, and obesity (Figure 4D). Heatmap shows that PPARs, TGFs, TNFs, ILs, collagen, and apoptosis-inducing gene expressions were significantly downregulated in isorhamnetin-treated hAECs (Figure 4E). On the other hand, insulin receptors, lipoprotein lipases, and apoptosis inhibitors were significantly upregulated in isorhamnetin-treated hAECs.

Our targeted microarray data analysis of isorhamnetin-treated hAECs also confirmed the potential of isorhamnetin in regulating biological functions related to DM and its associated complications.

## 6. Bioavailability and Intestinal Absorption of Isorhamnetin Aglycone and Its Glycosylated Derivatives

With the presence of various categories of flavonoids in nature, it is interesting to analyze the presence of these compounds in the body. When we talk about the metabolism of isorhamnetin and its availability in the human body after consumption, we are talking about the origin, the metabolism and the transport. This is based on an observation made by chromatography of some flavonoids such as flavonols in human serum. Liquid chromatography-mass spectrometry provides insight into the bioavailability of certain flavonoids in their aglycone and glycoside forms in the body [49]. Mass spectrometry can be used to determine flavonoids in biological samples [185]. A study reported 23 mixed sulfate, methyl, glucuronide, and glucose derivatives of quercetin in both urine and plasma of human volunteers 1 h after ingestion of lightly fried red onions. This study detected glycosides of both quercetin and isorhamnetin in plasma [186].

Several factors play a role in the entry of nutrients through the digestive tract. For example, enzymes from the intestinal microbiota affect the entry of phenolic compounds. A study carried out on the ginkgo leaf extracts in a mice model showed the importance of gut microbiota on the bioavailability and absorption from the gastrointestinal tract of some bioactive molecules, especially isorhamnetin [187]. In this step, enzymes of gut microbiota produce flavonoid aglycones and a variety of ring fission products. Analyses of the whole blood samples indicated that the uptake of isorhamnetin was increased by antibacterial treatment, suggesting that gut microbiota enzymes have a negative effect on the pharmacokinetics of natural molecules, such as isorhamnetin. Antibacterial or probiotic consumption may increase the bioavailability of the glycoside form of isorhamnetin. In addition, the in vitro biotransformation rates and residence times of bioactive molecules differed between normal, diabetic, and diabetic nephropathy rats [188].

**Figure 5:**
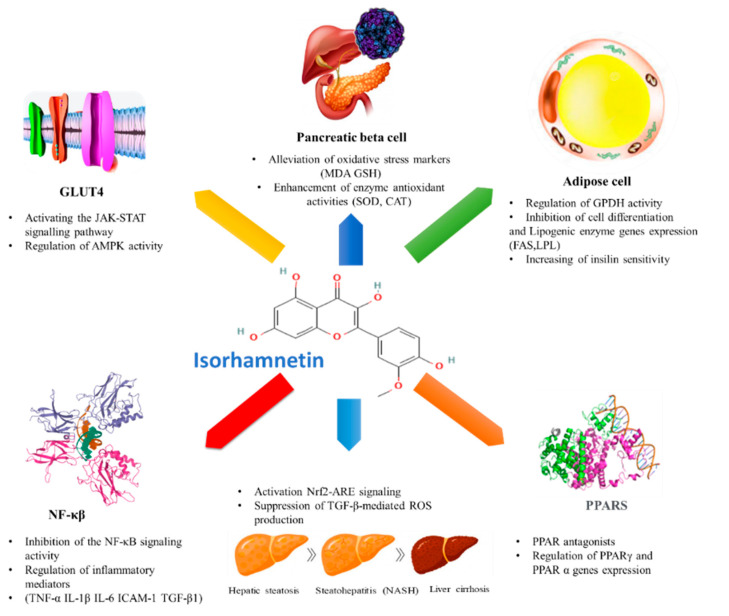
Anti-diabetic effects of isorhamnetin (NF-κβ and PPARS images were downloaded from Protein Data Bank (https://www.rcsb.org/, accessed on 10 November 2021), other images were freely downloaded from free picture database (https://fr.freepik.com/, accessed on 10 November 2021).

On the other hand, different membrane transporters control the transport of flavonoids, such as the sodium-dependent glucose transporter 1 (SGLT1) and the multidrug resistance-associated proteins 2 and 3 (MRP2 and MRP3) [189]. In this context, MRP transporters regulate the transcellular and the paracellular transport pathways of isorhamnetin [190]. Inside cells, the transport of isorhamnetin from the apical to the basal side was 6.8–9.3-fold higher. In Figure 5, the anti-diabetic effects of isorhamnetin are summarized.

## 7. Conclusions

Isorhamnetin is a phenolic compound of the flavonoid family, more precisely of the flavonols. Originally it is a quercetin molecule but has undergone methylation. Isorhamnetin is distributed in the plant kingdom in many wild and cosmetic plants. In addition, several medicinal plants produce this molecule and several studies have attested to its anti-diabetic effect among other biological activities. All these data thus show the interest of isorhamnetin in the therapeutic industry. From this perspective, it would be very interesting to explore the effect of isorhamnetin and its derivatives, isolated especially from natural resources, on metabolic disorders. It is also necessary to highlight and review the clinical studies performed in this context using flavonoid-rich fractions and natural products in order to avoid the impact of side effects caused by synthetic and chemical drugs.

## Figures and Tables

**Table 1:** Isorhamnetin resources cited in the current review.

Plant Sources	Extraction Methods	Action Modes	References
*Vaccinium vitis-idaea*	Fractionation guided 80% ethanol > ethyl acetate > water	Enhances muscle cell glucose uptake	[98]
*Nitraria retusa*	Maceration in water-ethanol solution	Modulation of lipogenesis–lipolysis balance	[17]
*Nitraria retusa*	Maceration in water-ethanol solution	3T3-L1 preadipocyte differentiation regulation	[18]
*Corchorus olitorius*	Soxhlet extraction with methanol, the residue from the above soluble extraction was hydrolyzed directly with 200 mL of 4 M NaOH solution. Then, the mixture was adjusted to pH 2 with concentrated HCl and the bound phytochemicals were extracted with ethyl acetate.	- α-Amylase inhibition - α-Glucosidase inhibition - Angiotensin I converting enzyme (ACE) inhibition - Degradation of deoxyribose	[95]
*Salicornia herbacea*	Fractionation guided ethanol under reflux > ethyl acetate ethyl acetate fraction was eluted on a silica gel chromatography by gradient elution with chloroform and methanol	- α-Glucosidase - Regulating the expression of ERK, PI3K, AKT, IRS-2, and PDX-1	[96]
*Eruca sativa*	Extraction with ethanol by the accelerated solvent extractor, at 50 °C, 1500 psi for 20 min	Activation of PPAR-α and suppression of inflammatory cytokines	[28]
*Oenanthe javanica*	Isorhamnetin was isolated from the aerial part of *O. javanica* and its purity was higher than 95.0%	Attenuation of fibrosis in rat hepatic stellate cells via inhibition of ERK signaling pathway	[99]

## Data Availability

Microarray data are deposited in the Gene Expression Omnibus (GEO) under Accession Number: GSE153149 (https://www.ncbi.nlm.nih.gov/geo/query/acc.cgi?acc=GSE153149, accessed on 24 November 2021).
